# Use of the speed achieved on the 6MWT for programming aerobic training in patients recovering from severe COVID-19: an observational study

**DOI:** 10.1080/07853890.2023.2179658

**Published:** 2023-03-07

**Authors:** María Fernanda del Valle, Jorge Valenzuela, Gabriel Nasri Marzuca-Nassr, Loretto Godoy, Mariano del Sol, Pablo A. Lizana, Máximo Escobar-Cabello, Rodrigo Muñoz-Cofré

**Affiliations:** a Servicio de Medicina Física y Rehabilitación, Hospital el Carmen, Maipú, Chile; b Departamento de Ciencias de la Rehabilitación, Facultad de Medicina, Universidad de La Frontera, Temuco, Chile; c Centro de Excelencia en Estudios Morfológicos y Quirúrgicos, Universidad de La Frontera, Temuco, Chile; d Laboratory of Morphological Sciences, Instituto de Biología, Pontificia Universidad Católica de Valparaíso, Valparaíso, Chile; e Laboratorio de Función Disfunción Ventilatoria, Departamento de Kinesiología, Universidad Católica del Maule, Talca, Chile; f Posdoctorado en Ciencias Morfológicas, Universidad de La Frontera, Temuco, Chile

**Keywords:** Coronavirus disease, pulmonary rehabilitation, exercise

## Abstract

**Introduction:**

Patients who suffered severe COVID-19 need pulmonary rehabilitation. Training may be prescribed objectively based on the maximum speed in the six-minute walk test. The objective of this study was to determine the effects of a personalized pulmonary rehabilitation program based on the six-minute walk test speed for post-COVID-19 patients.

**Methods:**

Observational quasi-experimental study. The pulmonary rehabilitation program consisted of 8 weeks of training, twice a week for 60 minutes per session of supervised exercise. Additionally, the patients carried out home respiratory training. Patients were evaluated by exercise test, spirometry and the Fatigue Assessment Scale before and after the eight-week pulmonary rehabilitation program.

**Results:**

After the pulmonary rehabilitation program, forced vital capacity increased from 2.47 ± 0.60 to 3.06 ± 0.77 L (*p* < .001) and the six-minute walk test result increased from 363.50 ± 88.87 to 480.9 ± 59.25 m (*p* < .001). In fatigue perception, a significant decrease was observed, from 24.92 ± 7.01 to 19.10 ± 7.07 points (*p* < .01). Isotime evaluation of the Incremental Test and the Continuous Test showed a significant reduction in heart rate, dyspnoea and fatigue.

**Conclusion:**

The eight-week personalized pulmonary rehabilitation program prescribed on the basis of the six-minute walk test speed improved respiratory function, fatigue perception and the six-minute walk test result in post-COVID-19 patients.

**KEY MESSAGES**
1. COVID-19 is a multisystem disease with common complications affecting the respiratory, cardiac and musculoskeletal systems.
2. The 6MWT speed-based training plan allowed for increased speed and incline during the eight-week RP program.
3. Aerobic, strength and flexibility training reduced HR, dyspnoea and fatigue in severe post-COVID-19 patients.

## Introduction

Worldwide, millions of people have suffered severe acute respiratory syndrome associated with COVID-19 [1]. Due to their severe respiratory symptoms, and in some cases acute respiratory difficulty, COVID-19 patients may require prolonged mechanical ventilation (MV). Consequently, they will require pulmonary rehabilitation (PR) during and after hospitalization [2,3]. Although COVID-19 generally presents as a respiratory infection, it is now known that it is a multisystemic disease with common complications affecting the respiratory and musculoskeletal systems [4].

Different criteria have been used to prescribe the intensity of aerobic exercise in different rehabilitation programs, for example determining ‘training zones’ through measurement of heart rate (HR) [5] or programming training workloads based on oxygen consumption [6]. The PR program must consider the morbid history and physiological parameters of each individual. Additionally, all PR programs must pay special attention to the duration, intensity, frequency and specificity of the exercise for each participant. This last part is essential to personalizing the training routine [2,4].

The 6-minute walking test (6MWT) has been used as an index suitable for determining the capacity for exercise of patients with respiratory problems [7], and the speed achieved is now being used to determine aerobic training loads [8].

A training intensity of around 80% of the maximum speed achieved on the 6MWT would generate clinical changes in exercise-related physiological responses. By contrast, exercise intensities below this workload will very likely not produce improvements in the exercise capacity. In addition to this, the 6MWT can determine intervention outcomes and predict survival rates. Thus, a change of more than 50 m in distance covered is clinically important in most pathological states. On the other hand, a distance covered less than 350 m is associated with higher mortality in chronic obstructive pulmonary disease (COPD). Therefore, increasing the distance covered has a positive impact on patients with respiratory pathologies [7,8]. Nevertheless, a scientific basis is needed to sustain the hypothesis that speed on the 6MWT can be used as a method to prescribe treadmill training [8], and this in conjunction with multimodal training (aerobic + strength + flexibility) may have benefits in patients with severe COVID. It is therefore necessary to continue examining physiological responses using this prescription method. Thus, the object of this study was to determine the effects of a personalized PR program based on the 6MWT speed for post-COVID-19 patients who have been on MV. The novelty of this study is to use the 6MWT speed to devise an individualized PR program. Second, to corroborate what is already known in other respiratory disorders, the 6MWT can be effective in determining changes in pulmonary abilities in severe COVID-19 patients.

## Methods

### Participants

Observational quasi-experimental study. Patients diagnosed severe COVID-19 were studied. The PR program is a routine procedure given to all patients recovering from Covid-19. However, for research purposes, patients who were part of the PR program were enrolled from January to April 2021. Inclusion criteria were: (a) diagnosis of COVID-19 through a positive PCR test, (b) MV required with orotracheal intubation, (c) medical discharge from hospital, (d) follow-up with cardiologist and normal electrocardiogram and (e) under health supervision (Hospital El Carmen in Maipú, Santiago, Chile). Patients who did not understand orders were excluded. This study is part of a large-scale project that aims to determine the effects of pulmonary rehabilitation on post-severe COVID-19 patients and already has previous publications. In this sense, the present research (i) details the incremental test (IT) and continuous test (CT) and their usefulness in programming training loads, (ii) shows how these tests also serve to determine the effects of training and (iii) are an objective tool that makes it possible to reprogram aerobic training loads during a RP program. The study was conducted according to the guidelines of the Helsinki Declaration and approved by the Scientific Ethics Committee of the Central Metropolitan Health Service (protocol code N° 392/2021). The participants read and signed an informed consent prior to admission to the PR program.

The reporting of the paper follows the STROBE guidelines (https://www.equator-network.org/reporting-guidelines/strobe/). This is to ensure effective and clear communication of all the important aspects of this research.

### Measurements

Measurements were taken before and after the eight weeks of the PR program. The evaluations were: exercise test, spirometry, maximum inspiratory pressure (MIP), fatigue evaluation.

### Exercise test

These tests were always performed in the following order: (i) 6MWT, (ii) IT and (iii) CT. Briefly, the 6MWT was used to measure the distance each participant walked in a corridor 30 m long and thus calculate their walking speed; this personalized the IT according to each participant’s condition. The resulting speeds and inclination applied in the IT were used to dose speeds and inclinations for aerobic training in the PR program The CT was used to evaluate the duration of the maximum speed obtained in the IT. In addition, to visualize the effect of the exercise on the participants, the isotime [9], HR, dyspnoea and fatigue results obtained in the IT and CT were compared before and after the PR program. Both the IT and the CT were conducted on a treadmill (Spirit CT800 212089^®^, Jonesboro, AR, USA). The stoppage criteria for the IT and CT were: dyspnoea or fatigue of ≥ 7 points on the Borg test, a pulse oximeter reading < 91% and/or exceeding 80% of the patient’s reserve heart rate [9].

#### 6-Minute walking test (6MWT)

Participants were instructed to walk as many meters as possible, at their own pace, in the 6 min assigned. The corridor used was marked every 1 m and was 30 m long [10]. Dyspnoea and lower limb fatigue were categorized with a modified Borg scale [11]. Pulse oximetry and HR were measured with a pulse oximeter before and after the test (Nonin 7500^®^, Nonin Medical, Minnesota, USA). The variables obtained were: distance covered in metres, HR, dyspnoea and fatigue. To calculate the predicted value, the predictive equations of Enright and Sherrill were used [12].

#### Incremental test (IT)

**Figure 1.**
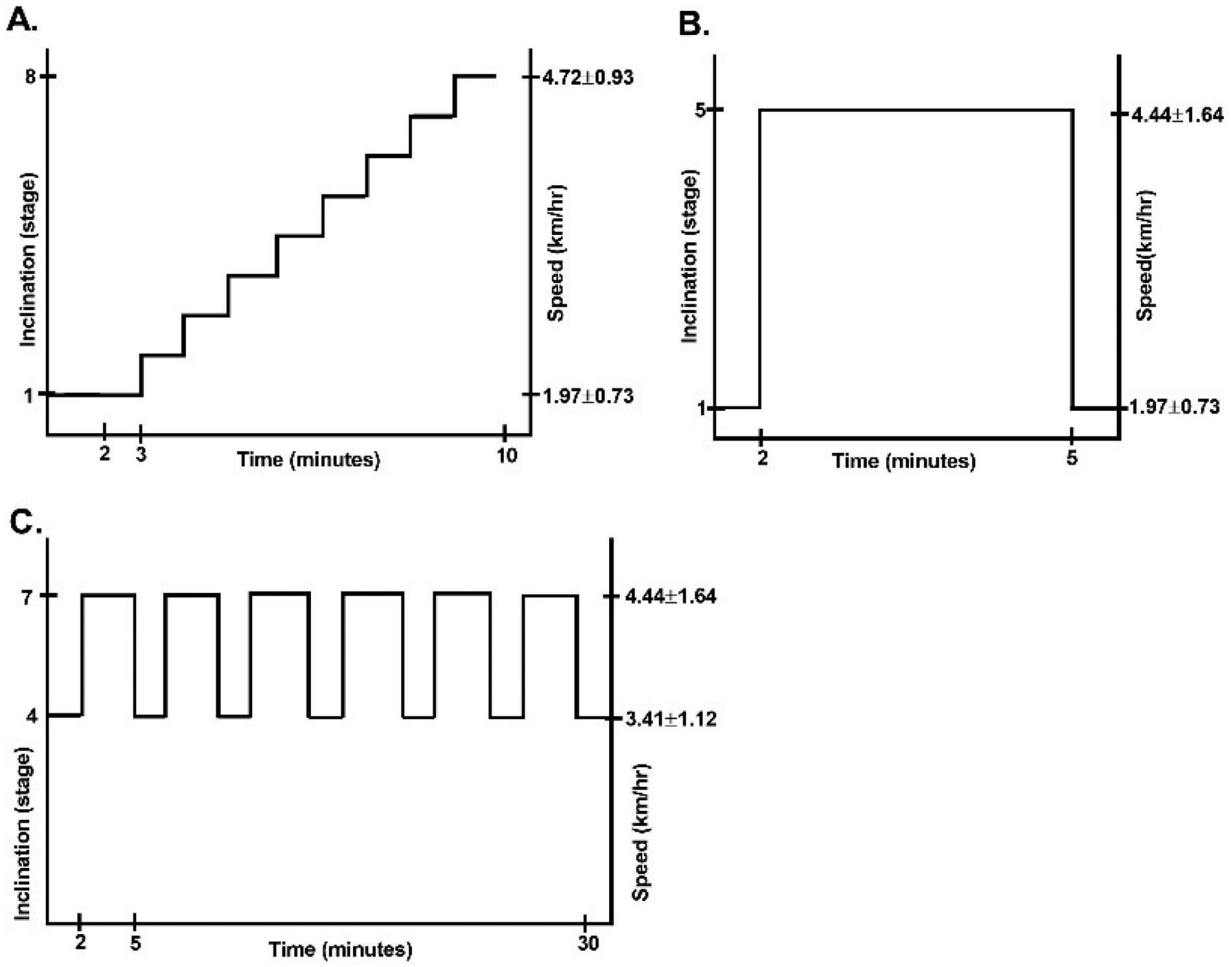
Diagram of the interval training strategy for severe COVID-19 survivors. (A) Incremental test, (B) continuous test and (C) interval training scheme. The lower and upper limits of inclination and speed corresponding to 60% and 80% of the maximum load of the incremental test.

The best speed reached on the 6MWT, i.e. the lowest time to cover the 30-m straight line in km/h, was used to determine the speeds in the IT. The treadmill was then set at a speed of 45% of the previously determined maximum, with a 1% incline as the initial load (Figure 1(A)). Every one minute the speed was increased by 15% and the incline by 1% [13,14]. HR, dyspnoea and fatigue were recorded.

#### Continuous test (CT)

This consisted of maintaining a constant speed and incline for the longest time possible. The fixed load was the greatest speed and incline obtained in the IT (Figure 1(B)) [13].

### Spirometry

Spirometric measurement was standardized according to the norms of the American Thoracic Society. It was taken in a 12 m^2^ cubicle, in the Physical Medicine service of the HEC, using a Medgraphics spirometer (CPFS/D USB 2.02, MGC Diagnostics Corporation, Minnesota, USA). The seated patient placed the pneumotachograph in their mouth and was asked to perform forced expiration starting from total lung capacity. The forced vital capacity (FVC) values and the expiratory volume in the first second (VEF_1_) were recorded, and the ratio between them was calculated [15]. The reference values used were from Quanjer et al. [16].

### Fatigue evaluation

This was measured with the Fatigue Assessment Scale (FAS). The FAS survey is self-administered and includes physical and mental fatigue dimensions. The answers are recorded on a 5-point Likert-type scale [17].

### Pulmonary rehabilitation program

The exercise sessions were done twice per week for eight weeks. Each session was divided into 30 min of aerobic exercise, 20 min of strength exercises and 10 min of recovery. The sessions were guided and supervised by a physiotherapist. Additionally, each patient carried out inspiratory muscle strength training at home [18].

In our previous publication, we describe the entire PR program. Briefly, the PR program consisted of an in-person part and another part with exercises done at home. In the in-person session, aerobic exercise was done on a treadmill. The aerobic work was personalized from the IT; the structure was as follows: speeds and inclines of 60% and 80% obtained in the IT were programmed at intervals of 2 and 3 min (Figure 1(C)). The criteria to stop training were the same as those used in the IT/CT, in addition to signs of lack of adaptation to exercise such as nausea, dizziness, pain, etc. The strength exercises were as follows: semi-squats and hip abductors were performed on the lower limbs with medium-resistance bands (green, Theraband, Hygenic Co., Akron, OH, USA). In the upper limbs, bilateral muscle chain exercises were performed with medium-resistance elastic bands (green, Theraband, Hygenic Co., Akron, OH, USA) on the biceps, triceps, trapezius, latissimus dorsi and abdominal muscles. Finally, flexibility was performed through stretching for each muscle group worked. During training, support was provided through 2 L of oxygen *via* nasal cannula, or according to the pulse oximetry of each patient.

In the home sessions, inspiratory muscle training was performed with a threshold valve (Philips Respironics, NJ, USA) IMT (Inspiratory Muscle Trainer), twice a day (morning and afternoon) for each day of the eight-week PR program [18].

### Statistical analyses

The results are presented as mean ± standard deviation and median (minimum – maximum). The statistics program used was STATA 16 (StataCorp, College Station, TX, USA). The normality of the data was determined *via* the Shapiro–Wilk test, followed by the Wilcoxon or Student’s t-test for paired samples depending on the data distribution. The patient’s condition before and after PR was compared by the variables of pulmonary function and 6MWT result, as well as the initial and final speeds of aerobic training. Finally, HR, dyspnoea and fatigue in the IT and CT were analysed. The significance level was set at *p* < .05. Also, the effect size was estimated using Cohen’s d and represented as d. An effect size <0.2 indicates no effect, 0.2–0.49 indicates small effect, 0.5–0.79 indicates medium effect and ≥0.8 indicates large effect.

## Results

**Figure 2.**
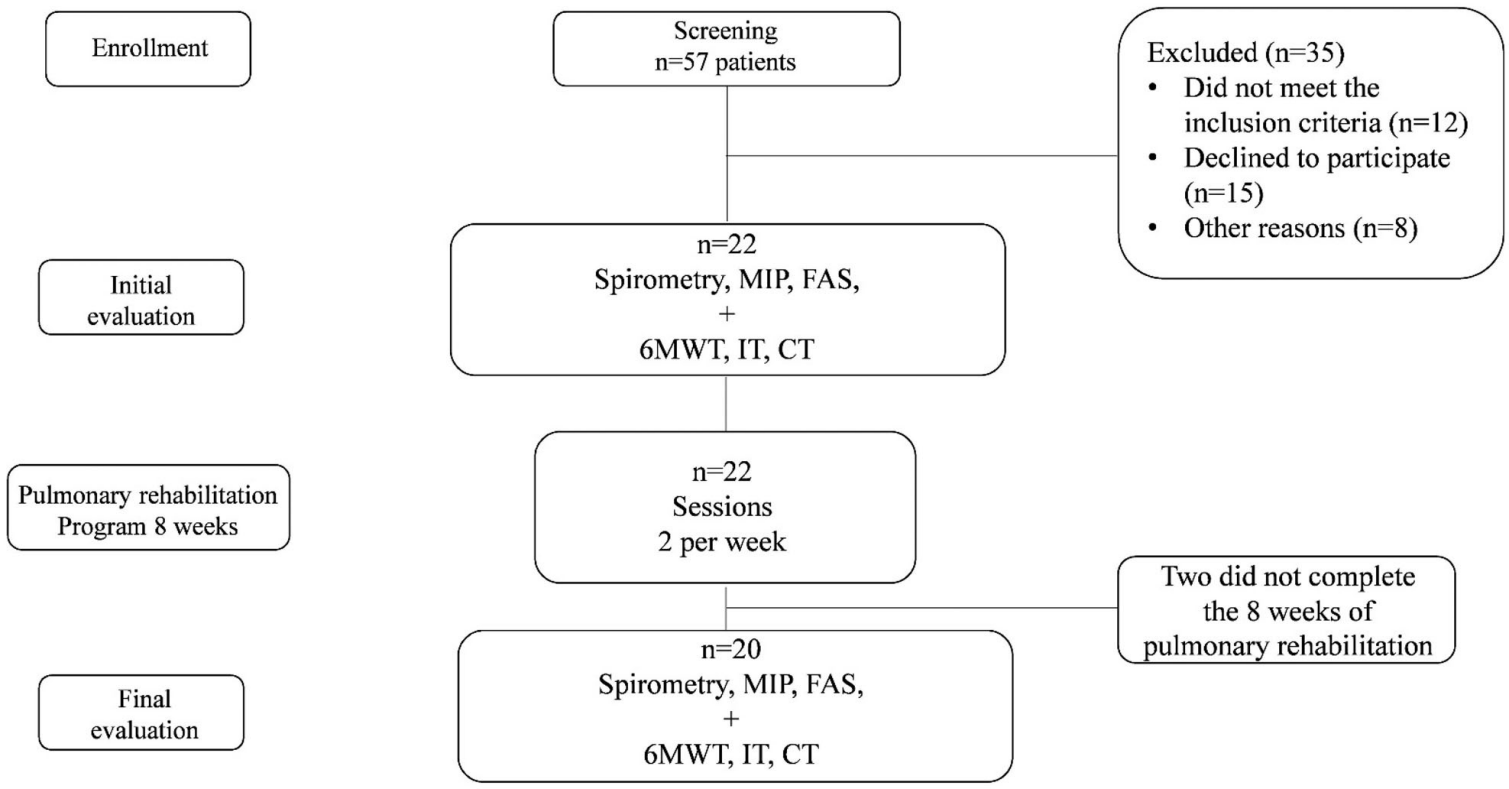
Study flow diagram. MIP: maximum inspiratory pressure; FAS: Fatigue Assessment Scale; 6MWT: 6-minute walking test; IT: incremental test; CT: continuous test.

Fifty-seven patients were screened; after review of the inclusion/exclusion criteria, 22 patients carried out the initial evaluation. During the eight weeks of the PR program, two patients withdrew voluntarily. Thus, 20 patients were included in the final analysis (Figure 2).

**Figure 3.**
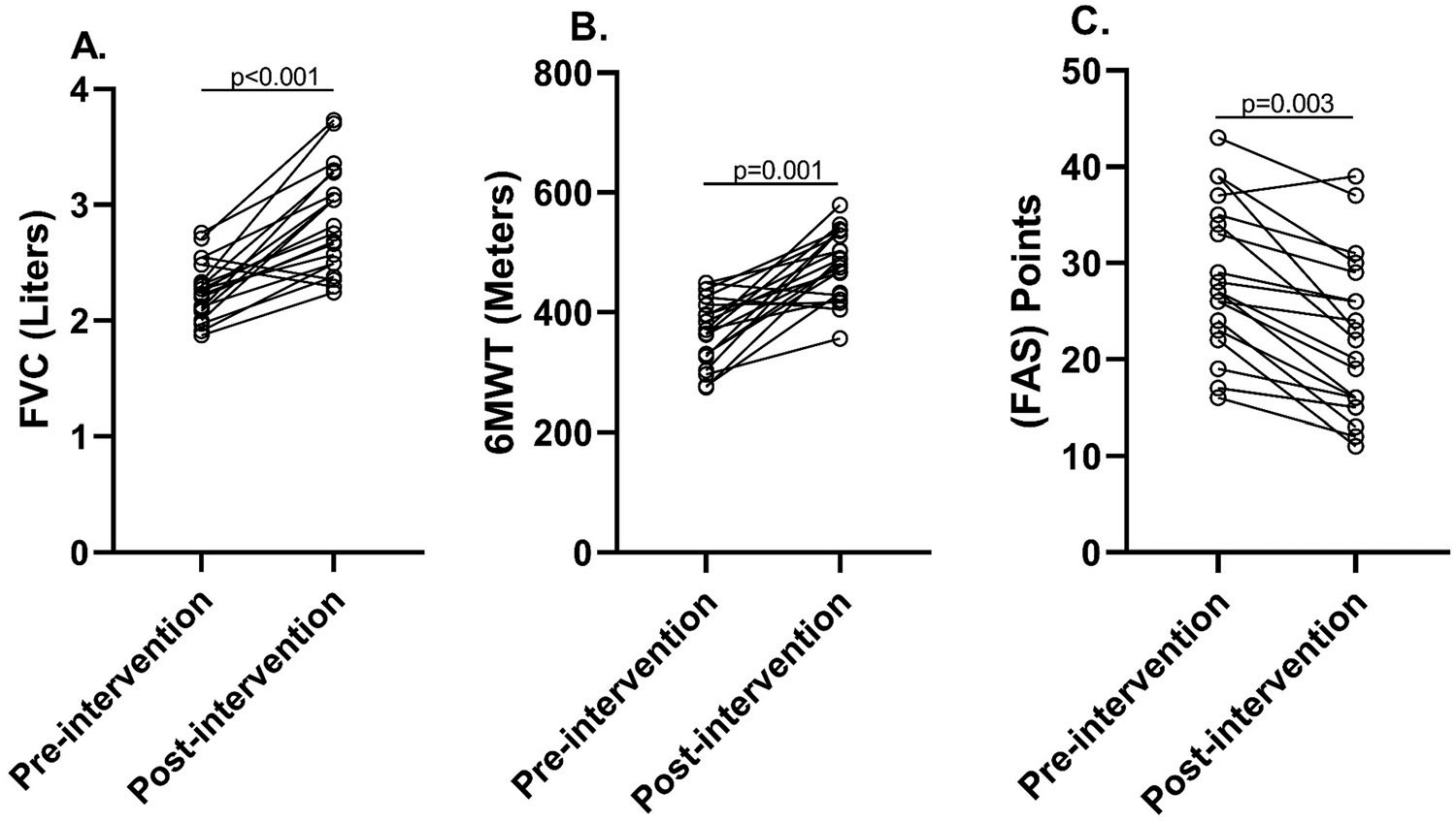
**(**A) Forced vital capacity; (B) distance covered on the 6MWT; (C) Fatigue Assessment Scale, total score. FVC: forced vital capacity; 6MWT: 6-minute walk test; FAS: Fatigue Assessment Scale.

**Table 1.** Anthropometric description and hospitalization data in severe post-COVID-19 patients (*n* = 20).

Variable	Mean
Age (years)	69 ± 6
Weight (kg)	75.2 ± 13.8
Height (cm)	159.4 ± 8.3
Female/male	9/11
BMI (kg/m^2^)	30.3 ± 2.6
Obesity (n/%)	11/55
DM (n/%)	9/45
Hypertension (n/%)	14/70
MV (days)	26.4 ± 23.5
Hospitalization time (days)	36.2 ± 22.0
Wait time to start PR (days)	85.0 ± 55.8

*Note:* BMI: body mass index; DM: diabetes mellitus; MV: mechanical ventilation; PR: pulmonary rehabilitation.

The basic characteristics of the patients are presented in Table 1. All the patients were aged over 60 years; 55% suffered obesity, 45% diabetes mellitus and 70% hypertension. The mean times on mechanical ventilation and hospitalization were 26.4 ± 23.5 and 36.2 ± 22 days, respectively. The average wait time for admission to the PR program was 85.0 ± 55.8 days.

FVC (*p* < .001; *d* = 0.855), FEV_1_, MIP and distance covered on the 6MWT (*p* < .001; *d* = 1.554) increased significantly following the eight-week PR program, while physical (*p* = .001; *d* = 0.474) and total (*p* = .003; *d* = 0.611) FAS decreased significantly (Figure 3). The workloads at the start and end of the program were as follows: at the baseline, 80% speed was 4.44 ± 1.64 km/h with an inclination of level 6 and 60% speed was 3.41 ± 1.12 km/h with an inclination of level 4; after the eight-week PR program (post-intervention), 80% speed was 6.25 ± 1.02 km/h with an inclination of level 9 and 60% speed was 5.00 ± 0.98 km/h with an inclination of level 7 (Table 2). Table 3 shows the progression of the 80% speed/inclination level and the 60% speed/inclination level during the eight weeks of the PR program.

**Table 2.** Pulmonary function and physical performance in severe post-COVID-19 patients (*n* = 20).

	Pre-intervention	CI 95%	Post-intervention	CI 95%	ES	*p* Value
*Espirometry*						
FVC (L)	2.47 ± 0.60	(2.10–2.83)	3.06 ± 0.77	(2.59–3.52)	0.855	<.001^t^
FVC (Pred)	3.10 ± 0.21		–			–
FVC (% predicted)	88.23 ± 9.73		–			–
FEV_1_ (L/s)	2.67 ± 0.85	(2.23–3.11)	2.91 ± 0.84	(2.36–3.22)	0.284	.028^t^
FEV_1_ (predicted)	2.92 ± 0.12		–			–
FEV_1_ (%predicted)	91.29 ± 23.55		–			–
FEV_1_/FVC	94.54 ± 7.23	(90.17–98.91)	85.85 ± 5.01	(82.82–88.87)	1.397	.015^t^
MIP (-cmH_2_O)	59.44 ± 24.56	(47.22–71.65)	71.20 ± 18.09	(62.21–80.19)	0.545	<.001^w^
*6-Minute walking test*						
Metres	363.5 ± 88.87	(309.8–417.2)	480.9 ± 59.25	(445.1–516.7)	1.554	<.001^w^
Predicted	465 ± 101.28		–			–
% Predicted	78.83 ± 26.24		–			–
HR	81.40 ± 8.50	(76.84–87.16)	73.60 ± 9.82	(73.96–86.34)	0.849	.001
S_pO2_	96.40 ± 1.75	(95.58–97.22)	96.50 ± 1.85	(95.63–97.37)	0.056	.733
Dyspnoea Borg scale	0 (0–3)	(0.12–1.27)	0 (0–3)	(0.03–0.86)	0.036	.437
*Fatigue Assessment Scale (FAS)*						
FAS mental (points)	13 (5–23)	(10.36–15.87)	11 (5–22)	(9.06–13.83)	0.631	.057^t^
FAS physical (points)	16 (11–22)	(14.15–17.63)	10 (6–23)	(9.34–13.85)	0.474	.001^w^
FAS total (points)	28 (16–43)	(25.11–32.89)	23 (5–39)	(17.96–26.81)	0.611	.003^t^
*Speed/inclination*						
60% (km/h)/(level)	3.41 ± 1.12/4	(2.01–4.82)	5.00 ± 0.98/7	(3.45–6.73)	1.511	.0001
80% (km/h)/(level)	4.44 ± 1.64/6	(2.42–5.61)	6.25 ± 1.02/9	(4.45–7.18)	1.325	.0002
Dyspnoea Borg scale (points)	4 (2–5)	(1.78–5.04)	5 (3–6)	(2.96–5.89)	0.229	.377
Fatigue Borg scale (points)	5 (4–7)	(2.79–7.11)	5 (3–7)	(1.67–6.76)	0.178	.999

*Note:* IC: confidence interval; ES: effect size *d_Cohen_*; FVC: forced vital capacity; FEV_1_: volume that has been exhaled at the end of the first second of forced expiration; MIP: maximum inspiratory pressure; cmH_2_O: centimetres of water; HR: heart rate; SpO_2_: pulse oximetry; 6MWT: 6-minute walk test; km/h: kilometres per hour; W: Wilcoxon; t: t Student.

**Table 3.** Speed and inclination progression of the aerobic training of the pulmonary rehabilitation program in severe post-COVID-19 patients (*n* = 20).

	Week 1	Week 2	Week 3	Week 4	Week 5	Week 6	Week 7	Week 8
80% Speed (km/h)/ inclination (level)	4.44 ± 1.64/6	4.44 ± 1.64/6	4.72 ± 0.93/7	4.72 ± 0.93/7	5.54 ± 1.53/8	5.54 ± 1.53/8	6.25 ± 1.02/9	6.25 ± 1.02/9
60% Speed (km/h)/ inclination (level)	3.41 ± 1.12/4	3.41 ± 1.12/4	3.77 ± 0.81/5	3.77 ± 0.81/5	4.51 ± 1.66/6	4.51 ± 1.66/6	5.00 ± 0.98/7	5.00 ± 0.98/7
Dyspnoea (points)	4 (2–5)	2 (1–4)	4 (3–6)	2 (1–4)	5 (3–7)	4 (3–5)	5 (3–7)	5 (3–6)
Fatigue (points)	5 (4–7)	4 (2–7)	5 (4–7)	4 (3–5)	6 (4–7)	4 (3–6)	5 (3–7)	5 (3–7)

*Note:* km/h: kilometres per hour.

Tables 4 and 5 compare the pre- and post-intervention values of HR, dyspnoea and fatigue in the IT and the CT, respectively. The tolerance to exercise at the same workload improved, as demonstrated by the significant reduction in HR, dyspnoea and fatigue from minute 3 onwards in both tests.

**Table 4.** Speed and inclination adjustments used in incremental treadmill exercise in severe COVID-19 survivors.

				HR (bpm)			Dyspnoea			Fatigue		
Incline	Time (min)	Speed (km/h)	*n* patients	Pre-int	Post-int	*p* Value	Pre-int	Post-int	*p* Value	Pre-int	Post-int	*p* Value
At rest	–	–	20	81.90 ± 13.33	77.43 ± 9.29	.096	0 (0–0)	0 (0–0)	>.999	0 (0–4)	0 (0–2)	.186
Warm-up	2	1.58 ± 0.73	20	95.00 ± 16.26	86.14 ± 11.00	.006	0 (0–0)	0 (0–0)	>.999	0 (0–5)	0 (0–4)	.149
1	3	1.97 ± 0.73	20	101.5 ± 15.16	89.43 ± 11.27	<.001	0 (0–3)	0 (0–0)	.006	0 (0–3)	0 (0–0)	.037
2	4	2.45 ± 0.70	20	108.1 ± 15.83	92.10 ± 12.93	<.001	2 (0–4)	0 (0–4)	.011	2 (0–5)	0 (0–4)	.001
3	5	2.94 ± 0.87	15	110.0 ± 17.54	94.86 ± 13.94	<.001	2 (0–7)	0 (0–4)	.005	3 (0–6)	0 (0–5)	<.001
4	6	3.41 ± 1.12	15	114.5 ± 19.10	98.90 ± 15.67	<.001	3 (0–7)	0 (0–4)	.002	3 (0–7)	0 (0–5)	<.001
5	7	3.87 ± 1.34	12	118.2 ± 22.26	102.7 ± 17.69	.001	3 (0–7)	0 (0–4)	<.001	3 (0–7)	0 (0–7)	<.001
6	8	4.09 ± 1.54	12	124.4 ± 22.18	110.2 ± 17.54	<.001	4 (0–7)	0 (0–4)	<.001	4 (0–7)	0 (0–7)	<.001
7	9	4.44 ± 1.64	10	125.3 ± 24.46	107.3 ± 21.13	<.001	5 (0–7)	0 (0–4)	<.001	6 (0–7)	0 (0–7)	<.001
8	10	4.72 ± 0.93	7	125.1 ± 22.66	108.6 ± 18.41	.001	5 (0–7)	1 (0–4)	<.001	5 (0–7)	0 (0–7)	<.001

*Notes:* HR: heart rate; bpm: beats per minute; km/h: kilometres per hour; pre-int: pre-intervention; post-int: post-intervention. Data are presented as mean ± SE; dyspnoea and fatigue data are presented as median (minimum – maximum). Statistical tests for paired samples were used.

**Table 5.** Speed and inclination adjustments used in continuous treadmill exercise in severe COVID-19 survivors.

				HR (bpm)			Dyspnoea			Fatigue		
Incline	Time (min)	Speed (km/h)	*n* patients	Pre-int	Post-int	*p* Value	Pre-int	Post-int	*p* Value	Pre-int	Post-int	*p* Value
At rest	–	–	20	86.77 ± 13.27	81.23 ± 10.93	.019	0 (0–0)	0 (0–0)	.400	0 (0–3)	0 (0–1)	.125
Warm-up	2	1.9 ± 0.75	20	101.9 ± 14.03	92.55 ± 14.69	<.001	0 (0–4)	0 (0–0)	.082	0 (0–3)	0 (0–1)	.005
4	3	4.09 ± 1.54	20	112.9 ± 19.75	97.86 ± 14.87	<.001	1 (0–4)	0 (0–3)	.003	1 (0–5)	0 (0–3)	.001
	4		20	120.5 ± 20.24	108.6 ± 17.40	<.001	2 (0–7)	0 (0–3)	<.001	3 (0–7)	0 (0–3)	<.001
	5		15	125.7 ± 22.21	112.8 ± 22.17	<.001	3 (0–7)	0 (0–4)	.001	3 (0–7)	0 (0–4)	.002
	6		15	130.9 ± 23.61	115.7 ± 23.01	<.001	4 (0–7)	0 (0–4)	<.001	4 (0–7)	0 (0–4)	.002
	7		12	131.7 ± 23.77	115.2 ± 23.00	<.001	5 (0–7)	0 (0–4)	<.001	5 (0–7)	0 (0–4)	<.001

*Notes:* HR: heart rate; bpm: beats per minute; km/h: kilometres per hour; pre-int: pre-intervention; post-int: post-intervention. Data are presented as mean ± SE; dyspnoea and fatigue data are presented as median (minimum – maximum). Statistical tests for paired samples were used.

## Discussion

The object of this study was to determine the effects of a personalized PR program based on the 6MWT speed for post-COVID-19 patients who have been on MV. The IT and CT were applied based on the patient’s maximum speed on the 6MWT, allowing the aerobic workloads to be personalized. The isotime evaluations showed a reduction in HR and perceptions of dyspnoea and fatigue. In addition to this, the results of pulmonary function and distance covered on the 6MWT coincide with those reported by this research group in previously published experiences [18]. In this respect, the present investigation; (i) describes the IT and the CT as assessment tools and useful for programming training loads, (ii) describes the increase in speed and inclination of aerobic training and (iii) includes the evaluation of fatigue perception, measured through the FAS, an important situation considering the musculoskeletal and psychological sequelae reported in post-COVID patients [2,3].

Aerobic training is an important part of PR [2]. Although workloads for this type of training have been prescribed in different ways, all agree on a ‘demand’ of ∼80% [5,6,8,19], which may be derived from the VO_2max_ (ergospirometry) [6] or from the maximum HR (Karvonen’s method) [5]. The present investigation proposes dosing the workload in aerobic training based on the maximum speed achieved on the 6MWT. The strategy used here, of interval work with a load of 80% for 3 min and 60% for 2 min, over a total time of 30 min, produced cardiopulmonary and physical improvements.

In cases of SARS-CoV-2 specifically, Daher et al. [20] studied the prevalence of organic and psychological dysfunctions in patients with COVID-19 six weeks after discharge from hospital. One of their results indicated a significant tendency in the patients to fatigue; this symptom limits their mobility, as reflected in a reduction in the distance covered on the 6MWT [20]. The results of the present investigation showed a significant increase in the distance covered on the 6MWT and a significant decrease in the total score and the physical dimension of the FAS. Consequently, we consider that the prescription of a training plan based on the patient’s maximum walking speed would have a positive functional impact on the physical fatigue of post-severe COVID-19 patients.

In this context, determining the maximum workload is critical for proper planning of the training activities. Porszasz et al. [21] implemented a protocol with an incremental workload on the treadmill based on computed equations. The authors observed the cardiopulmonary response and compared it with an IT on a cycle ergometer [21]. Their results showed that the treadmill protocol provided a similar linear profile in the workload to that of the cycle ergometer, which is the current standard. It is therefore valid to use the treadmill to evaluate subjects with low tolerance to exercise.

Although the results of the present investigation also show a sustained improvement in HR, dyspnoea and fatigue as the speed and inclination were increased, our methodology differed from that of Porszasz et al. [21]. The present study programmed the workloads in the incremental test based on the maximum speed achieved on the 6MWT [21]. This allowed us to increase the treadmill speed by 15% per minute on an individual basis; we conclude that implementing a ‘climb’ in speed and inclination on the treadmill is fundamental to achieving this linear increase in workload, the objective of all ramp tests [9,13].

In terms of the effect of walking as a training activity, McDermott et al. [14] compared the changes in the maximum distance walked on the treadmill with the distance covered on the 6MWT in response to a therapeutic intervention in participants with peripheral arterial damage. To do this, they carried out a randomized clinical trial that included supervised exercise on the treadmill, walking at home and a placebo for peripheral arterial damage. Exercise at home improved the distance covered on the 6MWT by 43.2 m, while supervised exercise on the treadmill improved the distance covered on the 6MWT by 25.0 m (a difference in the means of +18.2 m in favour of exercise at home). The group that received treadmill training presented an improvement of 141.3 m more than the home training group in the maximum distance covered on the 6MWT on a treadmill. The investigators concluded from this result that supervised treadmill training amplified the improvement in the distance achieved on the treadmill due to training for the test and did not act as a training strategy [14]. This contrasts with the results of the present investigation, which showed a significant increase in the distance covered on the 6MWT after the PR training period. This may be attributed to the different training strategies, since McDermott et al. [14] proposed a continuous workload of 2 mph (≈ 3.2 k/h) for 15 min increasing to 40 min by the eighth week [14]. The methodology proposed in the present investigation programmed aerobic training loads based on the maximum speed on the 6MWT, which were increased as the patient adapted. Reprogramming is fundamental to applying the overload necessary to generate physical improvement.

Use of the IT made it possible to dose the aerobic training in response to each patient’s individual needs. Eston et al. [13] evaluated the accuracy of estimating the VO_2peak_ from the HR:VO_2_ ratio, recorded in a submaximal test on the treadmill. They concluded that a test with incremental loads based on the perception of effort indices is a valid method for predicting VO_2peak_ in young individuals and from middle to old age at different levels of activity and physical condition [13]. This is consistent with the observations of the present study, where the strategy of dosing the exercise based on IT performance made it possible to implement aerobic training for 30 min with reprogramming of speeds that increased during the eight weeks of the PR program. Complementary to the IT, the present study implemented a CT which allowed us to indirectly evaluate this phenomenon and also contributed to determining the high workload blocks in interval training for each patient.

With respect to the usefulness of the IT and CT in programming the workload, Zainuldin et al. [8] examined exercise intensity in COPD patients who trained by walking at 80% of their mean speed on the 6MWT. To do this, each patient was asked to walk round a 32 m oval track continuously for 10 min (Walk-10) at a constant speed adjusted to 80% of their mean speed on the 6MWT. The mean intensity of Walk-10 was 77 ± 13% VO_2peak_ and the stationary state in VO_2_ was achieved after 4 min of Walk-10. Therefore walking at 80% of the mean speed of the 6MWT is a high but tolerable intensity of exercise, which probably results in benefits for most people with COPD [8]. Although the aerobic training structure used in the present investigation includes 3 min with a load of 80% of the speed achieved on the 6MWT, we believe that the following three points, as a complement to strength training, will help to improve the distance covered on the 6MWT: (i) the training speed is calculated from the maximum speed on the 6MWT, (ii) the training period lasts for 30 min and (iii) continuous, progressive readjustment of the workload during the eight weeks of the PR program.

This study has limitations which must be specified: (i) the sample homogeneity of participants, (ii) lack of a control group might create a statistical bias in our conclusions as we cannot compare the effectiveness of this program versus usual care since all patients received the PR program, (iii) direct measurement of the VO_2max_ could have helped to assess objective behaviour in the IT and CT and (iv) due to the ongoing pandemic at the time of this study, the average waiting time to enter the PR program was almost three months, which may have had a negative influence on the results obtained.

In conclusion, aerobic training programmed based on the maximum speed achieved on the 6MWT proved to be feasible and applicable. The training proposal also reduced HR, dyspnoea and fatigue under a similar workload as well as improving exercise tolerance in patients post-severe COVID-19. We suggest the use of the 6MWT maximum speed as a low-cost alternative, combined with the CT and IT for exercise training and complementing them with strength and flexibility training.

## Data Availability

The data used in this research is available. Please send request to Dr. Rodrigo Muñoz-Cofré.
